# Eumycetoma Caused by *Madurella pseudomycetomatis* in a Captive Tiger (*Panthera tigris*)

**DOI:** 10.3390/jof8121289

**Published:** 2022-12-09

**Authors:** Margherita Orlandi, Giuseppe Giglia, Patrizia Danesi, Piero Laricchiuta, Francesca Abramo

**Affiliations:** 1 Private Veterinary Laboratory “MyLav”, Passirana di Rho, 20017 Milan, Italy; 2 Department of Veterinary Medicine, University of Perugia, 06126 Perugia, Italy; 3 Division of Pathology, Department of Biomolecular Health Sciences, Faculty of Veterinary Medicine, Utrecht University, 3584CL Utrecht, The Netherlands; 4 Istituto Zooprofilattico Sperimentale delle Venezie, 35020 Legnaro, Italy; 5 Zoosafari, Fasano, 72015 Bari, Italy; 6 Department of Veterinary Sciences, University of Pisa, 56124 Pisa, Italy

**Keywords:** animal, eumycetoma, histology, *Madurella*, phaeohyphomycosis, tiger

## Abstract

A captive-kept adult male tiger presented with a large cutaneous and subcutaneous mass on the thigh with a fistula. During sedation, multiple nodules were detected and samples for a histopathological exam were collected. Histologically, granulomatous panniculitis and dermatitis were seen around dense aggregates of pigmented fungal hyphae, and a diagnosis of phaeohyphomycosis was made; considering the clinical features, it was classified as a eumycotic mycetoma. This is a rarely reported subcutaneous fungal infection in humans and animals, caused by dematiaceous fungi. Clinically, it is characterized by tumefaction, fistulous sinus tracts, and the formation of macroscopically visible grains. In the literature, only a few infections in wild felids have been reported. In this case, Fontana–Masson staining better showed pigmentation and panfungal PCR and sequencing identified *Madurella pseudomyectomatis* (OP623507) as the causative agent. Systemic therapy with oral administration of itraconazole was planned, but the patient died during the first period of treatment. The animal was not submitted for post-mortem examination. Visceral dissemination of the agent cannot be excluded. To the authors’ knowledge, this is the first report of eumycotic mycetoma by *Madurella pseudomycetomatis* in a captive tiger.

## 1. Introduction

Eumycotic mycetoma or eumycetoma represents a chronic subcutaneous fungal infection. It is clinically characterized by tumefaction, fistulous sinus tracts, and the formation of macroscopically visible black or pale grains. Saprophytic pigmented (dematiaceous) and nonpigmented fungi are the cause of the eumycetoma. Infection is usually acquired through traumatic implantation or direct open-wound contamination [1]. Histologically, when dematiaceous fungi are involved, mycotic lesions are referred to as chromomycosis. Additionally, lesions are further classified as chromoblastomycosis if only round, thick-walled structures (sclerotic bodies) are identified, or as phaeohypomycosis if yeast-like structures and hyphae are observed. On the contrary, when non-dematiaceous fungi are involved, lesions are classified as hyalohyphomycosis [2]. Pigmentation seen in dematiaceous fungi is associated with melanin synthesis by the cell wall and is thought to act as a virulence factor [3].

Among the organisms that cause eumycetoma with black grains in both humans and animals are *Alternaria* spp., *Bipolaris* spp., *Cladophialophora* spp., *Curvularia* spp., *Exophiala* spp., and *Madurella* spp. [4,5,6,7].

Different to other fungal diseases, such as cryptococcosis, dermatophytosis, and sporotrichosis, chromomycosis is not listed among the most important zoonotic fungal agents [8,9]. Nonetheless, given the pathogenicity of dematiaceous fungi, they should be considered significant pathogens for humans and animals.

In the literature, there are numerous case reports of cutaneous and visceral infections caused by dematiaceous fungi in cats, while in wild captive-kept felids only a few have been reported [5,6,10,11,12,13].

Specifically, *Cladophialophora bantiana* was isolated from an abscess in the spinal cord of a snow leopard, [11] *Cladosporium* spp. from a cerebral lesion in a lion, [12] and *Curvalaria* spp. in disseminated cutaneous and visceral lesions in a Sumatran tiger [13].

Herein, we describe the first case of a eumycetoma caused by *Madurella pseudomycetomatis* in a captive-kept tiger in Italy.

## 2. Detailed Case Description

A male adult tiger (*Panthera tigris*) (age estimated around 8 years), weighing about 180 kg, born and held at Zoo Safari Park in Fasano (Southern Italy), underwent clinical examination for a cutaneous mass that had developed within 2 months on the thigh. All clinical procedures were performed after the tiger was sedated with 0.01 mg/kg of medetomidine and 3 mg/kg of ketamine. During the anesthesia, fluid therapy (10 mL kg/h) was administered, and blood samples were obtained from the jugular vein and submitted for hemato-biochemical examination.

Reference intervals reported in the Zoological Information Management Systems (ZIMS) database were used for comparisons [14].

The blood panel showed a marked leukocytosis (67.91 × 10^3^/µL; range: 5.4–16.9) characterized by monocytosis (3390/µL; range: 70–840) and neutrophilia, with band neutrophils (4754/µL; range: 0–4430) and segmented neutrophils (59,081/µL; 3700–13,900).

The tiger had elevated uremia (273 mg/dL; range: 16.6–53), moderate creatinemia (5.90 mg/dL; range: 1.0–4.3), altered urea/creatinine ratio (46.27; range: 5.3–26.3), and mildly elevated creatinine phosphokinase (CPK) (816 IU/L; range: 75–688). The other parameters examined were within the normal range. Serum protein electrophoresis showed a polyclonal hypergammaglobulinemia.

The hematobiochemical and electrophoretic findings, associated with absence of systemic symptoms, were compatible with pre-renal impairment/dehydration and a chronic inflammatory state. Mildly elevated CPK was probably due to muscular damage caused by anesthetic injection through a remote delivery system.

The mass was 10 cm wide, focally ulcerated, and had poorly defined margins. During sedation, multiple variably ulcerated and soft cutaneous nodules were identified all over the body. Given the age of the animal, the main differential diagnoses included a metastatic neoplastic process and a systemic infection process (deep mycosis and bacterial granulomas).

Two skin biopsies 5 × 1 cm and 4 × 1.5 cm in diameter, including skin and subcutis, were fixed in a 10% buffered formalin solution and submitted for histopathological examination. After adequate fixation, the samples were processed for routine histopathology, embedded in paraffin, and 5 µm-thick sections were cut and stained with hematoxylin and eosin (HE).

**Figure 1:**
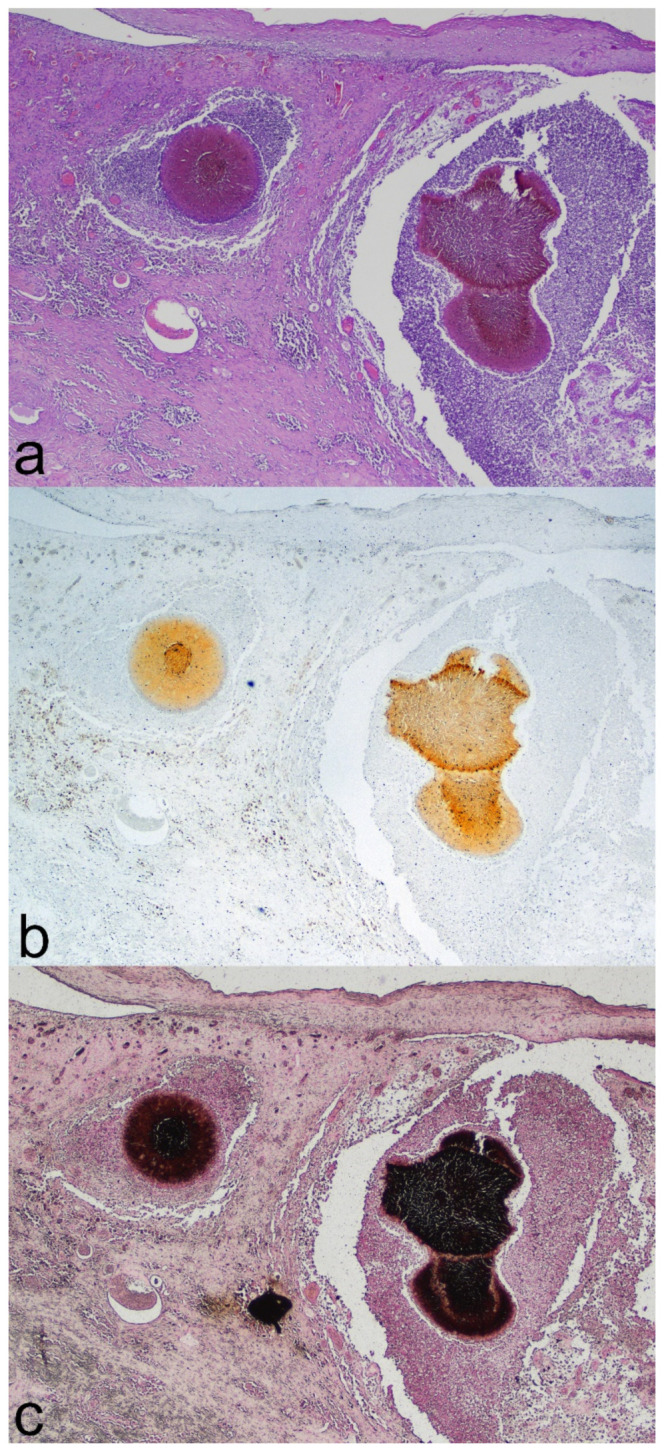
Skin histopathology. (**a**) In the superficial dermis, two individual nodules composed of pyogranulomatous reactions centered on dark-pink fungal aggregates (HE stain). (**b**) Serial unstained section demonstrating the light-brownish pigmentation of the fungal aggregates. (**c**) Serial section stained with Fontana–Masson stain showing highly melanized fungal aggregates.

**Figure 2:**
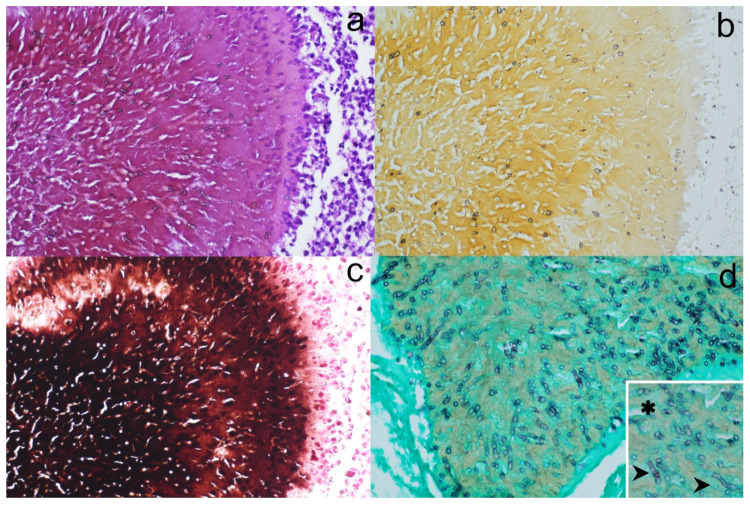
Histopathology and histochemistry of granules. HE stain (**a**) shows the presence of hyphal aggregates within the granules; unstained (**b**) and Fontana–Masson-stained (**c**) sections show the presence of a faint brownish pigment (**b**) or a very dark melanized background within the granules; Grocott staining (**d**) allows better definition of hyphal characteristics; short hyphae (arrows) and cystic dilations (asterisks) are indicated in the inset.

On histopathology examination, a cutaneous and subcutaneous non-neoplastic lesion was detected, covered by an irregular hyperplastic epidermis lining the opening of a fistulous tract. The mass, with ill-defined margins, was composed of multiple individual-to-coalescent nodules with different shapes, measuring from 0.3 to 1.3 mm, which after HE staining appeared a dark pinkish-brownish color (Figure 1a). Each of the nodules was composed of 2–3 aggregates containing large amounts of hyphal elements (Figure 2a). The hyphae were embedded in a radiating, thin, amorphous eosinophilic material (Splendore–Hoeppli reaction). Fungal aggregates were circumscribed by an inflammatory infiltrate composed of macrophages, epithelioid cells, scattered multinucleated giant cells, and small mature lymphocytes, separated by an oedematous vascularized stroma.

With viewing of unstained sections, fungal aggregates showed a faint yellow-to-brownish background (Figure 1b and Figure 2b). To confirm the nature of the pigment, a Fontana–Masson stain was performed; the pigmentation in the fungal wall and background stained positive and therefore was confirmed as melanin (Figure 1c and Figure 2c).

To better highlight the fungal morphology, a Grocott–Gomori methenamine silver (GMS) stain was performed. The fungal aggregates stained positively, the hyphae were 3–5 μm in diameter and 8–12 μm in length, usually organized in short septated hyphae, with occasional irregular branching and rare terminal cystic dilations 5–6 μm in diameter (consistent with chlamydoconidia) (Figure 2d).

The morphological diagnosis was a severe, multifocal-to-coalescing, granulomatous dermatitis and panniculitis with intralesional pigmented fungal hyphae; thus, a histological diagnosis of a phaeohyphomycosis was made.

**Figure 3:**
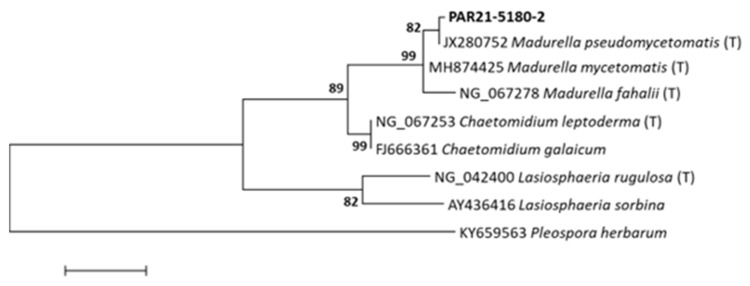
Phylogenetic tree based on portions of the 28S LSU sequences of various members of the order Sordariales. In boldface, PAR21-5180-2 was the sequence produced in our study. The *Pleospora herbarum* sequence was used as an outgroup. The maximum likelihood method was used to construct the tree. Bootstrap values shown at the main nodes represent the probabilities based on 1000 replicates. (T) = type strains.

To identify the causative agent, DNA was extracted from 10 µm-thick sections (*n* = 3) and amplified by SYBR^®^ Green real-time PCR assay using in-house primers 26S 56-73 for 5′-TAACGGCGAGTGAASCGG-3′ and 26S 329-311 rev 5′-TACTTGTKCGCTATCGGTC-3′ targeting a portion of the D1/D2 region of 28S rRNA (amplicon length: 230/260 bp), as reported previously [15]. Sequencing reactions of PCR amplicons were performed from both ends. Alignment was performed with Clustal W integrated into MEGA v6.0. *Madurella pseudomycetomatis* was identified by comparing the obtained sequences in the GenBank database using BLAST. *Madurella pseudomycetomatis* (OP623507) from this study showed 99% similarity with the *M. pseudomycetomatis* type strain (JX280752). Molecular phylogeny was performed on the 28S LSU rRNA sequence dataset. The rooted tree was constructed, including *Madurella pseudomycetomatis* from this study and other Sordariales species (*Madurella, Chaetomidium, and Lasiosphaeria* species) available from the GeneBank database (Figure 3). A sequence of *Pleospora herbarum* was used as an outgroup. Phylogenetically, the *M. pseudomycetomatis* from this study grouped with the *M. pseudomycetomatis* clade and was monophyletic with related species in the *Madurella* clade.

At the time of histological diagnosis, an antimycotic therapy was planned. Itraconazole, a systemic fungicidal drug, was administered daily for 3 weeks (one capsule containing 100 mg of the active substance) with only little clinical improvement. Before the end of the treatment, the tiger was found dead and was not submitted for post-mortem examination.

## 3. Discussion

To the best of the authors’ knowledge, this is the first report of *Madurella pseudomycetomatis* infection causing eumycetoma in a captive tiger. The tiger presented with multiple cutaneous masses all over the body, most of which showed ulceration, fistulization, and swelling, which is the typical presentation for this disease.

Histopathological findings, with the identification of fungi characterized by the pigmentation of the wall, allowed us to classify the lesions as a eumycetoma with melanin-producing fungi. Hematoxylin and eosin (HE) staining is generally sufficient to achieve a diagnosis [16]. However, when present only in a small amount, pigmentation can be overlooked by routine HE staining. Very easily in the case herein described, pigmentation was documented by simply avoiding any staining of the section; by directly viewing under the microscope an unstained section, it was possible to detect the yellowish color of the fungal aggregates. It has been reported that fungi can produce two types of defensive pigments, melanins and carotenoids. Among fungal melanins, different types have been documented: eumelanin, DHN-melanin, pyomelanin, pheomelanin and GHB melanin [17]. Eumelanin gives a black-brown color to cells, whereas pheomelanin has a yellow-red pigmentation [18]. Since both pheomelanin and carotenoids have yellowish pigmentations, the melanin-producing capacity of the fungus was further confirmed by Fontana–Masson staining [19,20]. From both unstained and stained sections, it appeared that the yellowish melanin was abundant in the extracellular location, different to other fungi in which melanin can be found within the cell wall or in the cytoplasm [21]. Melanin is considered a virulence factor, since it prevents the penetration of antifungal drugs [22].

In addition, GMS staining allows for a better evaluation of fungal morphology, except for pigmentation [2]. In our case, GMS enhanced the observation of septation and of chlamydoconidia.

Although the histopathology allowed a straightforward diagnosis, it failed to identify the causative organism at the species level.

Fungal culture and/or molecular techniques are needed to identify the specific species involved. In fact, some studies have revealed that the phenotypic features of fungi might lack correlations with molecular identifications [23].

Based on the availability of formalin-fixed paraffin-embedded block (FFPE) material, sections were submitted for a DNA-based molecular approach, which led to the identification of *Madurella pseudomycetomatis* as the causative agent. To date, only rare cases of *M. pseudomycetomatis* infection in the human literature have been reported; it is considered a neglected disease, endemic in tropical and subtropical countries [24,25,26]. In veterinary medicine, only a recent report by Albanese et al. (2022) in a dog described a subcutaneous mass on the inner thigh [27]. To date, no reports of *M. pseudomycetomatis* infection have been reported in domestic or wild felids.

Infections by dematiaceous fungi are occasionally associated with immunosuppression, both due to an underlying cause and iatrogenic, due to corticosteroid administration [1]. In this tiger, no underlying deficit in the immune system was detected.

The course of the disease is usually slow, and the persistence after inoculation is dependent on the efficiency of the immune system of the host and/or the pathogenicity of the microorganism. For this reason, the development of lesions from the initial exposure is variable, making it difficult to define the moment and the source of the infection, as in this case [28]. This tiger was born in the zoo; hence, the most likely source of infection was a wound contamination from the environment of the enclosure. It is known that eumycetoma-causative agents are found prevalently in tropical and subtropical areas but also in the soil of other part of the world, and their distribution might depend on climatic changes [29].

To date, there are no large studies on the treatment of eumycetoma and phaeohyphomycosis in felids. The proposed therapy was based on the few case reports on domestic cats and in the human literature. Different therapeutic protocols are available, such as surgical excision or prolonged administration of itraconazole, ketoconazole, posaconazole, and intravenous amphotericin B [30].

Single lesions are more commonly removed by an aggressive surgical excision, while a prolonged administration of itraconazole is the treatment of choice in cases of multiple lesions or single lesions in areas where they are difficult to remove. Amphotericin and posaconazole are preferred in case of disseminated disease [1,10]. As an important note, phaeohyphomycosis antifungal drugs might have reduced effects, due to the presence of melanin [3,31].

Of the cases of chromomycosis in wild felids reported in the literature, only in one case was a therapy established. The therapy included intravenous amphotericin B lipid complex in combination with oral administration of itraconazole. No clinical improvement was observed, and subsequent euthanasia was performed [13].

Given the presence of multifocal lesions, surgery was not taken into consideration in the case herein reported. Therefore, an antifungal systemic therapy with itraconazole was set, with no apparent clinical improvement and sudden death reported before the end of the treatment. Unfortunately, the animal was not submitted for a post-mortem examination. For this reason, the evaluation of the efficacy of the therapy and possible systemic dissemination as the cause of death were not ascertainable. Although not frequent, visceral involvement causing a worsening of clinical condition can occur, as previously reported in a Sumatran tiger, and this can also be hypothesized for the case herein described [13].

## 4. Conclusions

In conclusion, to the best of the authors’ knowledge, this is the first case report of infection by *Madurella pseudomycetomatis* in a captive-kept tiger in Italy, for which the diagnosis was achieved by light microscopy and PCR. The disease showed progression with multiple skin nodules, and medical therapy was demonstrated to be ineffective. A visceral dissemination was suspected as the cause of death.

## Data Availability

Not applicable.
